# Severity of obesity and management of hypertension, hypercholesterolaemia and smoking in primary care: population-based cohort study

**DOI:** 10.1038/jhh.2015.23

**Published:** 2015-03-26

**Authors:** H P Booth, A T Prevost, M C Gulliford

**Affiliations:** 1 Department of Primary Care and Public Health Sciences, King's College London, London, UK

## Abstract

Obesity and obesity-associated cardiovascular risk are increasing worldwide. This study aimed to determine how different levels of obesity are associated with the management of smoking, hypertension and hypercholesterolaemia in primary care. We conducted a cohort study of adults aged 30–100 years in England, sampled from the primary care electronic health records in the Clinical Practice Research Datalink. Prevalence, treatment and control were estimated for each risk factor by body mass index (BMI) category. Adjusted odds ratios (AOR) were estimated, allowing for age, gender, comorbidity and socioeconomic status, with normal weight as reference category. Data were analysed for 247 653 patients including 153 308 (62%) with BMI recorded, of whom 46 149 (30%) were obese. Participants were classified into simple (29 257), severe (11 059) and morbid obesity (5833) categories. Smoking declined with the increasing BMI category, but smoking cessation treatment increased. Age-standardised hypertension prevalence was twice as high in morbid obesity (men 78.6% women 66.0%) compared with normal weight (men 37.3% women 29.4%). Hypertension treatment was more frequent (AOR 1.75, 1.59–1.92) but hypertension control less frequent (AOR 0.63, 0.59–0.69) in morbid obesity, with similar findings for severe obesity. Hypercholesterolaemia was more frequent in morbid obesity (men 48.2% women 36.3%) than normal weight (men 25.0% women 20.0%). Lipid lowering therapy was more frequent in morbid obesity (AOR 1.83, 1.61–2.07) as was cholesterol control (AOR 1.19, 1.06–1.34). Increasing obesity category is associated with elevated risks from hypertension and hypercholesterolaemia. Inadequate hypertension control in obesity emerges as an important target for future interventions.

## Introduction

Obesity is a growing global health concern, with a rapid increase being observed in morbid obesity. Excess body weight is associated with an increased cardiovascular risk and earlier onset of cardiovascular morbidity.^1^ It is well established that obesity is associated with activation of both the sympathetic nervous system and the renin–angiotensin system contributing to the emergence of hypertension.^2^ Epidemiological studies have demonstrated the importance of the level and duration of obesity as risk factors for cardiovascular disease.^3^ Improved preventive medical care to detect, treat and control of risk factors in overweight and obese patients is therefore of particular importance.^4^ Smoking, high blood pressure (BP) and elevated serum cholesterol levels represent major cardiovascular risk factors^5^ that are routinely managed in primary care settings. Deficits in risk factor detection, treatment and control are well recognised. The ‘rule of halves' has been applied to hypertension management, which suggests that half of hypertension may be detected, with half of these treated and half controlled.^6^ In the UK general population, hypertension detection, treatment and control have improved over time with closer to ‘two-thirds' now being detected, treated or controlled.^6^ Some studies suggest that hypertension may be less well controlled in obese patients,^7, 8, 9^ but few studies have been sufficiently large to analyse patients with severe and morbid obesity as separate groups. Management of hypercholesterolaemia and smoking at different levels of obesity have been less studied.^7^ In the UK, a population-based system of primary care, in which patients remain registered with the same general practice for many years, enables the health care system to potentially address these important concerns. This study aimed to compare the diagnosis and treatment of smoking, hypertension and hypercholesterolaemia, and control of hypertension and hypercholesterolaemia, according to body mass index (BMI) category by using the electronic health records of a large population in primary care.

## Methods

### Study design and participants

CPRD is a prospectively collected database of primary care electronic health records, from ~680 UK general practices with 5.5 million currently registered patients.^10^ For the present study, data were sampled from CPRD general practices in England that participate in the data linkage scheme. A random sample of patients registered in CPRD, aged 30 years or older, was drawn. This provided an initial cohort of 300 006 patients. These analyses evaluated records during 2011 and we included all 247 653 participants, with 122 903 men and 124 750 women, who contributed person-time in 2011. Ethical approval for the study was given by the CPRD Independent Scientific Advisory Committee (ISAC 09_085 & 14_056).

### Outcomes and covariates

BMI records were evaluated and BMI was also calculated from weight and height records where appropriate. Measurements from up to 3 years previously were analysed.^11^ Patients were classified by BMI using the categories: underweight, BMI<18.5 kg m^−^^2^; normal weight, 18.5–24.9 kg m^−2^; overweight, 25.0–29.9 kg m^−2^; obese, 30.0–34.9 kg m^−^^2^; severe obesity, 35.0–39.9 kg m^−^^2^; and morbid obesity, ⩾40 kg m^−^^2^. Patients with missing BMI status were included in the analyses as a separate category.

Smoking status was ascertained by using clinical records of smoking habits (including cigarettes, pipe, cigar and unspecified smoking) together with records of advice, referrals to stop smoking services and drug prescriptions for nicotine replacement therapy, as reported in detail previously.^12^ Participants not recorded as smokers or ex-smokers were classed as non-smokers.^12^ Records of smoking cessation advice, referrals or prescriptions for nicotine replacement therapy were used to indicate treatment for smoking cessation. ‘Control' of smoking was not evaluated as clinical records of quitting and relapse may be incomplete.

Hypertension was defined as a BP reading of ⩾140/90 mm Hg or treatment with antihypertensive drugs.^13^ Antihypertensive drugs were categorised by using the ‘ABCD' classification, including: A, angiotensin-converting enzyme inhibitors and angiotensin receptor blocking drugs; B, beta blockers; C, calcium channel blockers; and D, thiazide diuretics.^13^ Hypercholesterolaemia was defined as a total cholesterol measurement >5 mmol l^−1^ or treatment with lipid-lowering drugs. Treatment of each condition was assessed based on the prescription of antihypertensive drugs or lipid-lowering drugs. Successful control was defined as a BP <140/90 mm Hg, or total cholesterol ⩽5 mmol l^−1^. Socioeconomic deprivation was assessed by using the Index of Multiple Deprivation rank based on patient postal code and divided into quintiles for England.^14^

### Data analysis

Age-standardised prevalences for smoking, hypertension and hypercholesterolaemia were calculated for using the European Standard Population for reference.^15^ Multivariable logistic regression models were used to compare the proportion receiving treatment across BMI categories. In order to evaluate whether treatment of risk factors differed depending on the need for primary or secondary prevention, sensitivity analyses were performed by excluding patients with comorbid coronary heart disease (CHD), stroke or type 2 diabetes from analyses. Models were adjusted for age and gender, prevalent CHD, stroke, diabetes and deprivation quintile.

## Results

The initial cohort comprised 300 006 patients. For the present analyses, we included all 247 653 participants, with 122 903 men and 124 750 women, who contributed person-time in 2011. The mean age was 56.0 (s.d. 14.3) for men and 57.7 (s.d. 15.4) for women. There were 153 308 (62%) with BMI values recorded in the preceding three years, including 29 257 (19%) with simple obesity, 11 059 (7.2%) having severe obesity and 5833 (3.8%) with morbid obesity. Among patients with known smoking status, 25.5% of men and 20.8% of women were current smokers.

Age-standardised prevalences for smoking, hypertension and hypercholesterolaemia by gender and BMI category are presented in Table 1. Current smoking was highest in underweight patients, at 56.4% (95% confidence interval (CI) 51.8–61.1) in men and 37.2% (34.5–39.8) in women. Smoking prevalence declined with increasing BMI category, reaching 21.6% (19.6–23.6) in morbidly obese men and 19.7% (18.3–21.0) in women. The prevalence of hypertension and hypercholesterolaemia rose with increasing BMI category. Hypertension was found to affect 78.6% (76.6–80.7) of morbidly obese men and 66.0% (64.5–67.4) of morbidly obese women, while hypercholesterolaemia was prevalent in 48.2% (45.9–50.4) of morbidly obese men and 36.3% (34.9–37.7) of women. The proportion with each risk factor was generally higher in men than women in the same BMI category.

**Figure 1:**
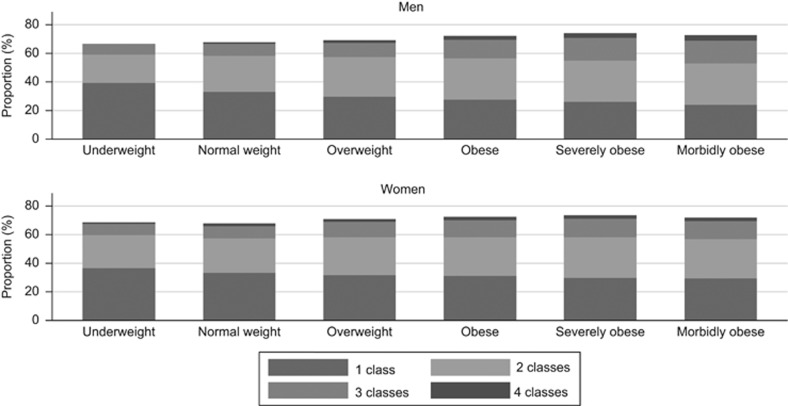
Use of antihypertensive drugs by BMI category. Proportion of hypertensive patients receiving different classes of antihypertensive drugs.

Figure 1 presents the proportion of patients with hypertension receiving antihypertensive medication by BMI category. The proportions receiving one, two, three or four classes of antihypertensive medications are also shown. As BMI category increased a higher proportion of hypertensive patients received antihypertensive drug treatment. The proportion of patients prescribed one class of drug decreased slightly in higher BMI categories, from 47% of normal weight men to 37% of morbidly obese men. However, there was only marginal increase in the proportion receiving three or four classes of antihypertensive drugs at higher levels of obesity. In women, the values were 49 and 48%, respectively.

**Figure 2:**
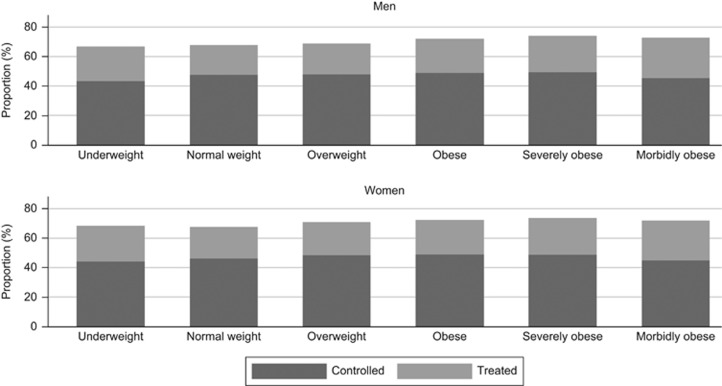
Treatment and control of hypertension by BMI category in 2011. Bars represent the proportion of hypertensive patients who received treatment and those with a blood pressure measurement of <140/90 mm Hg.

**Figure 3:**
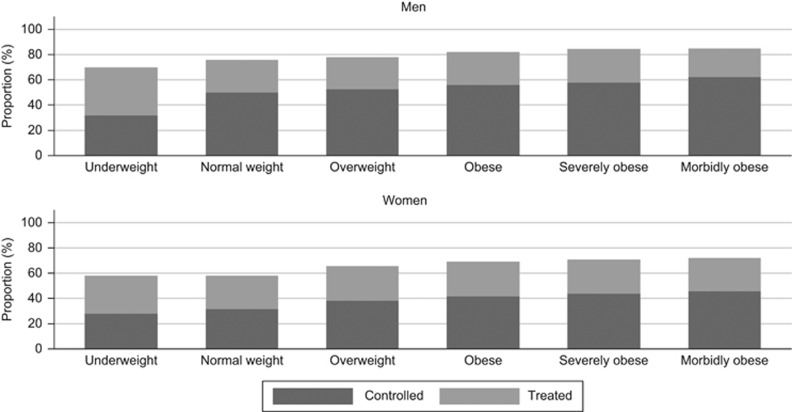
Treatment and control of hypercholesterolaemia by BMI category in 2011. Bars represent the proportion of patients with hypercholesterolaemia who received the treatment and those with a total cholesterol measurement of ⩽5 mmol l^−1^.

Figure 2 presents the proportion of patients with treated hypertension whose BP was controlled. As BMI category increased, the proportion with controlled hypertension did not increase in proportion to those treated, and tended to decrease in morbid obesity (Table 3). However, for hypercholesterolaemia (Figure 3) both the proportion treated and controlled increased with an increasing BMI category (Tables 2 and 3).

Table 2 presents adjusted odds ratios (AOR) for the association of BMI category with risk factor treatment. The odds of receiving treatment for each of the three risk factors increased with increasing BMI category. The effect of increasing BMI category was greatest for treatment of hypercholesterolaemia, where obesity was associated with odds of 1.59 (1.48–1.72, *P*<0.001) compared with normal weight, and morbid obesity with an AOR of 1.83 (1.61–2.07, *P*<0.001). The absolute difference in proportion treated was 10% (Table 2). For treatment of hypertension, the AOR for morbid obesity was 1.75 (1.59–1.92, *P*<0.001) compared with normal weight, with an absolute difference of 4%. For smoking cessation therapy the AOR was 1.32 (1.11–1.58, *P*=0.002), with an absolute difference of 6%. Patients with missing BMI status were less likely to be treated. Greater socioeconomic deprivation was associated with a greater likelihood of receiving treatment for hypertension (AOR 1.16, 95% CI 1.07–1.26, *P*=0.001 most deprived vs least deprived quintile) and hypercholesterolaemia (AOR 1.28, 95% CI 1.08–1.53, *P*=0.005), but not for smoking. Presence of co-morbidities was associated with greater odds of treatment for each of the risk factors. To evaluate whether obesity was associated with risk factor management in the absence of cardiovascular comorbidity, we additionally excluded data for patients with established CHD, stroke and type 2 diabetes and observed similar associations.

Increasing BMI category was associated with lower hypertension control (Table 3). Morbidly obese patients with treated hypertension had an AOR of 0.63 (0.57–0.69, *P*<0.001) compared with normal weight patients and 7% fewer had their BP controlled. The odds of controlled hypercholesterolaemia were greater in morbidly obese patients compared with normal weight (AOR 1.19, 1.06–1.34, *P*=0.005), with 7% more having the cholesterol values controlled (Table 3). Control of hypertension and hypercholesterolaemia was lower in those with missing BMI (AOR 0.65, 0.60–0.72, *P*<0.001). A diagnosis of CHD, stroke and diabetes was associated with greater odds of controlled hypertension and hypercholesterolaemia, whilst socioeconomic deprivation was associated with worse cholesterol, but not hypertension, control. Additional analyses were performed, excluding patients with existing diabetes CHD or stroke. These analyses showed that the associations of hypertension control with BMI category were similar in a primary prevention cohort but cholesterol control was no longer associated with BMI category.

## Discussion

### Summary of key findings

Obesity is associated with more frequent hypertension and hypercholesteroalemia; higher rates of treatment for smoking, hypertension and hypercholesterolaemia, but worse control of hypertension. Separate estimates were presented for severe and morbid obesity, showing that associations were graded according to level of obesity. Control of hypertension was negatively associated with BMI category, with morbidly obese patients less likely to have controlled BP than those who were of normal weight. Evaluation of the number of classes of antihypertensive drugs prescribed showed that maximal antihypertensive therapy was not utilised in a high proportion of patients. Further research is needed to evaluate the use of different classes of drugs, and different agents within classes, in order to identify optimal hypertensive therapy for obese patients. Conversely, obesity was associated with better control of total cholesterol. The absolute magnitude of effects was modest but these small differences may be of public health importance when a frequent condition is managed in a large population.

### Comparison with the literature

Obesity is known to be associated with greater cardiovascular risk^16^ and a higher incidence of cardiovascular events.^17^ Hypertension is associated with increased morbidity and mortality from major cardiovascular diseases.^18^ The incidence^17^ and prevalence^19^ of hypertension are greater as BMI category increases. In the Framingham cohort,^20^ obese individuals were more likely to receive treatment for hypertension but there were no differences in hypertension control. Less consistent associations have been reported for treatment and control of hypercholesterolaemia. In the Framingham study, hypercholesterolaemia, based on a cut-point of 6.2 mmol l^−1^, was not as strongly associated with obesity as was hypertension,^17^ but rates of lipid-lowering therapy were higher in the obese participants.^20^ However, several factors potentially confound this relationship. Access and adherence to lipid-lowering therapy may sometimes depend on the socioeconomic deprivation category.^21^

### Strengths and limitations

The study had the strength of a very-large sample, drawn from a nationally representative population of eligible participants. The size of the sample enabled us to obtain precise estimates for participants with severe and morbid obesity. However, we acknowledge that analysis of clinical records for key measures suffered from several limitations. Nearly 40% of participants did not have recent BMI values recorded, consistent with the infrequent monitoring of BMI in primary care.^11^ The distribution of BMI category was generally similar to those observed in national survey data^22^ but confounding by indication may arise if BMI is more likely to be measured in patients who are ill or have elevated risk factor values. Obese patients may be more likely to have their BP and lipid levels documented compared with their normal weight peers.^23^ Recording of measurements may be associated with treatment. Errors in BP measurement may be greater in more obese people, if a correctly fitting BP cuff is not used. The use of different drug classes may vary by obesity category requiring further in-depth analysis. We reported on total cholesterol values because lipoprotein fractions are less frequently recorded into electronic health records. However, use of LDL:HDL ratios might lead to different interpretations if HDL levels are lower in obesity. We used a cut-point of 5 mmol l^−1^ to define hypercholesterolaemia and control, but lower treatment targets are sometimes recommended. We were not able to analyse data for some relevant confounders including physical activity, alcohol consumption, educational level, ethnicity, family history or duration of hypertension. We conducted sensitivity analyses to evaluate the relevance of cardiovascular comorbidity for the measures evaluated, but we acknowledge that there may be other relevant co-morbidities, such as type 1 diabetes, which might be present and lower frequency.

### Implications for future research and practice

The results of this large population-based study reveal that treatment and control of cardiovascular risk remain sub-optimal in both obese and non-obese patients. However, the status of three major modifiable risk factors for cardiovascular disease differs according to weight status. Smoking is generally less frequent as BMI increases but obese smokers are more likely to receive smoking cessation therapy. Obese individuals more frequently have hypercholesterolaemia, are more likely to receive treatment, and tend to be slightly better controlled. However, while hypertension is more frequent in obesity, and there is evidence of a greater intensity of antihypertensive treatment as BMI category increases, rates of hypertension control remain less satisfactory. These associations were broadly consistent in men and women but further research is required to explore possible gender differences in the impact of obesity. The results identify the increase in obesity as a major obstacle to improved hypertension management, perhaps now threatening the continued decline in cardiovascular disease. While weight reduction may be associated with improved BP and reduced cardiovascular risk^16^ non-surgical weight-loss interventions presently have generally limited long-term effects.^24, 25^ Although the pathophysiological mechanisms of hypertension in obesity have been well studied,^2^ the reasons why hypertension is more difficult to control in obese subjects deserve more research. Improved intervention strategies, and optimal therapeutic interventions, for hypertension in obesity may then be developed.


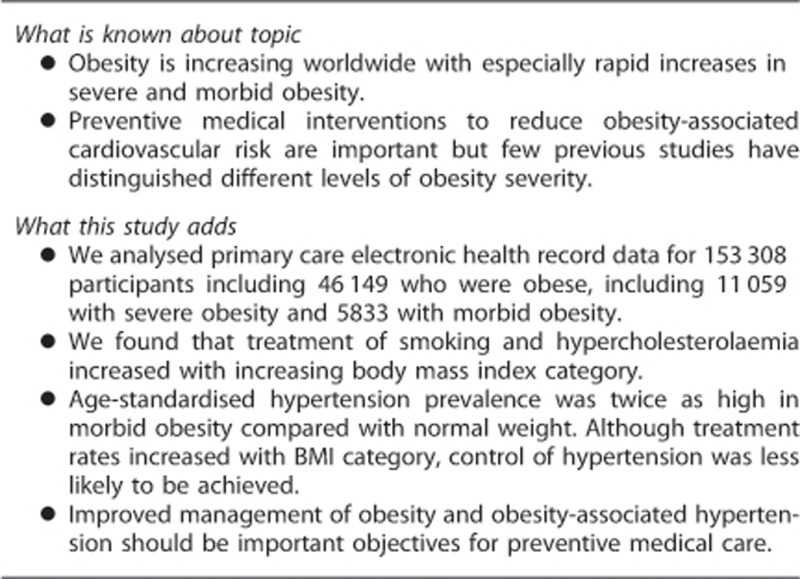


## Figures and Tables

**Table 1:** Age standardised prevalence (%) of smoking, hypertension and hypercholesterolaemia in men and women in 2011

*BMI category*	N	*Smoking*		*Hypertension*		*Hypercholesterolaemia*	
		*Number of smokers*	*ASR (95% CI)*	*Number with BP records*	*ASR (95% CI)*	*Number with cholesterol records*	*ASR (95% CI)*
*Men*							
Underweight	608	298	56.4 (51.8–61.1)	271	30.7 (26.8–34.5)	164	18.2 (14.9–21.4)
Normal weight	17 697	5045	33.2 (32.4–34.0)	8848	37.3 (36.5–38.0)	6300	25.0 (24.4–25.6)
Overweight	30 130	6425	24.4 (23.8–25.0)	18 278	49.5 (48.9–50.1)	13 385	33.6 (33.0–34.1)
Obese	14 683	3029	23.5 (22.6–24.3)	10 357	61.1 (60.2–62.0)	7379	39.9 (39.1–40.7)
Severely obese	4486	884	21.5 (20.1–22.9)	3470	71.0 (69.5–72.6)	2343	44.4 (42.9–46.0)
Morbidly obese	1927	402	21.6 (19.6–23.6)	1576	78.6 (76.6–80.7)	1025	48.2 (45.9–50.4)
Missing BMI	53 372	13 540	24.9 (24.5–25.3)	19 761	38.1 (37.7–38.5)	8544	17.0 (16.7–17.3)
*Women*							
Underweight	1935	587	37.2 (34.5–39.8)	877	26.6 (24.6–28.5)	542	15.8 (14.1–17.5)
Normal weight	30 012	6677	23.5 (23.0–24.0)	11 559	29.4 (28.9–29.9)	7858	20.0 (19.6–20.4)
Overweight	26 777	5344	21.9 (21.3–22.5)	13 801	38.9 (38.4–39.5)	9424	25.9 (25.5–26.4)
Obese	14 574	2868	21.9 (21.2–22.7)	8706	49.2 (48.4–50.0)	5801	29.9 (29.2–30.6)
Severely obese	6573	1206	19.7 (18.6–20.7)	4286	57.3 (56.1–58.5)	2645	33.3 (32.3–34.4)
Morbidly obese	3906	745	19.7 (18.3–21.0)	2741	66.0 (64.5–67.4)	1566	36.3 (34.9–37.7)
Missing BMI	40 973	8186	21.1 (20.7–21.5)	16 228	33.0 (32.6–33.4)	7817	15.3 (15.0–15.7)

Abbreviations: ASR, age-standardised prevalence % BMI, body mass index; CI, confidence interval.

**Table 2:** Multivariable logistic regression models for the treatment of smoking, hypertension and hypercholesterolaemia

	N	*Treated (%)*	*OR (95% CI)*	P*-value*
*Smoking*^a^				
Underweight	885	95 (11)	0.74 (0.58, 0.95)	0.017
Normal weight	11 722	1648 (14)	1.00	—
Overweight	11 769	1830 (16)	1.08 (0.99, 1.18)	0.070
Obese	5897	993 (17)	1.16 (1.05, 1.29)	0.005
Severe obesity	2090	374 (18)	1.20 (1.04, 1.39)	0.016
Morbid obesity	1147	227 (20)	1.32 (1.11, 1.58)	0.002
Missing BMI	21 726	1571 (7)	0.51 (0.35, 0.93)	<0.001
				
*Hypertension*^a^^b^				
Underweight	1148	781 (68)	0.72 (0.62, 0.84)	<0.001
Normal weight	20 394	13 787 (68)	1.00	—
Overweight	32 087	22 343 (70)	1.21 (1.16, 1.27)	<0.001
Obese	19 064	13 744 (72)	1.50 (1.41, 1.58)	<0.001
Severe obesity	7757	5707 (74)	1.75 (1.62, 1.88)	<0.001
Morbid obesity	4319	3107 (72)	1.75 (1.59, 1.92)	<0.001
Missing BMI	36 000	13 322 (37)	0.45 (0.41, 0.50)	<0.001
				
*Hypercholesterolaemia*^a,^^b^				
Underweight	706	424 (60)	0.70 (0.58, 0.84)	<0.001
Normal weight	14 158	9291 (66)	1.00	—
Overweight	22 809	16 574 (73)	1.35 (1.28, 1.43)	<0.001
Obese	13 180	9957 (76)	1.59 (1.48, 1.72)	<0.001
Severe obesity	4998	3852 (77)	1.83 (1.66, 2.02)	<0.001
Morbid obesity	2591	1981 (76)	1.83 (1.61, 2.07)	<0.001
Missing BMI	16 361	7426 (45)	0.70 (0.64, 0.78)	<0.001

Abbreviations: BMI, body mass index; CI, confidence interval; N, represents the number of patients with the risk factor; OR, odds ratio.

a Adjusted for age, gender, CHD, stroke, type 2 diabetes and socioeconomic status.

b Adjusted for smoking status.

**Table 3:** Multivariable logistic regression models for the control of hypertension and hypercholesterolaemia in treated patients

		*Hypertension*				*Hypercholesterolaemia*		
	N	*Controlled (%)*	*OR (95% CI)*^a^^b^	P*-value*	N	*Controlled (%)*	*OR (95% CI)*^a^	P*-value*
*BMI category*								
Underweight	781	501 (64)	0.84 (0.71–0.98)	0.031	424	202 (48)	0.63 (0.51–0.78)	<0.001
Normal weight	13 787	9505 (69)	1.00	—	9291	5637 (61)	1.00	—
Overweight	22 343	15 341 (69)	0.93 (0.88–0.98)	0.004	16 574	10 645 (64)	1.10 (1.04–1.16)	<0.001
Obese	13 744	9251 (67)	0.84 (0.79–0.89)	<0.001	9957	6487 (65)	1.09 (1.03–1.16)	0.004
Severe obesity	5707	3759 (66)	0.78 (0.73–0.84)	<0.001	3852	2520 (65)	1.08 (1.00 1.18)	0.059
Morbid obesity	3107	1912 (62)	0.63 (0.59 0.69)	<0.001	1981	1343 (68)	1.19 (1.06 1.34)	0.005
Missing BMI	13 322	6746 (51)	0.54 (0.51 0.58)	<0.001	7426	3073 (41)	0.65 (0.60 0.72)	<0.001

Abbreviations: BMI, body mass index; CI, confidence interval; N, number receiving treatment; OR, odds ratio.

a Controlling for age, gender, co-morbidities, smoking status and socioeconomic status.

b Also controlling for number of antihypertensive drug classes.
